# Clustering-local-unique-enriched-signals (CLUES) promotes identification of novel regulators of ES cell self-renewal and pluripotency

**DOI:** 10.1371/journal.pone.0206844

**Published:** 2018-11-06

**Authors:** Chao Wu, Yang Jiao, Manli Shen, Chen Pan, Guo Cheng, Danmei Jia, Jing Zhu, Long Zhang, Min Zheng, Junling Jia

**Affiliations:** 1 Life Sciences Institute and Innovation Center for Cell Signaling Network, Zhejiang University, Hangzhou, Zhejiang, PRC; 2 Beijing Ming-tian Genetics Ltd., Beijing, PRC; 3 Collaborative Innovation Center for Diagnosis and Treatment of Infectious Diseases, Zhejiang University, Hangzhou, Zhejiang, PRC; 4 State Key Laboratory for Diagnosis and Treatment of Infectious Diseases, The First Affiliated Hospital, Zhejiang University, Hangzhou, Zhejiang, PRC; Chuo University, JAPAN

## Abstract

**Background:**

Key regulators of developmental processes can be prioritized through integrated analysis of ChIP-Seq data of master transcriptional factors (TFs) such as Nanog and Oct4, active histone modifications (HMs) such as H3K4me3 and H3K27ac, and repressive HMs such as H3K27me3. Recent studies show that broad enrichment signals such as super-enhancers and broad H3K4me3 enrichment signals play more dominant roles than short enrichment signals of the master TFs and H3K4me3 in epigenetic regulatory mechanism. Besides the broad enrichment signals, up to ten thousands of short enrichment signals of these TFs and HMs exist in genome. Prioritization of these broad enrichment signals from ChIP-Seq data is a prerequisite for such integrated analysis.

**Results:**

Here, we present a method named Clustering-Local-Unique-Enriched-Signals (CLUES), which uses an adaptive-size-windows strategy to identify enriched regions (ERs) and cluster them into broad enrichment signals. Tested on 62 ENCODE ChIP-Seq datasets of Ctcf and Nrsf, CLUES performs equally well as MACS2 regarding prioritization of ERs with the TF’s motif. Tested on 165 ENCODE ChIP-Seq datasets of H3K4me3, H3K27me3, and H3K36me3, CLUES performs better than existing algorithms on prioritizing broad enrichment signals implicating cell functions influenced by epigenetic regulatory mechanism in cells. Most importantly, CLUES helps to confirm several novel regulators of mouse ES cell self-renewal and pluripotency through integrated analysis of prioritized broad enrichment signals of H3K4me3, H3K27me3, Nanog and Oct4 with the support of a CRISPR/Cas9 negative selection genetic screen.

**Conclusions:**

CLUES holds promise for prioritizing broad enrichment signals from ChIP-Seq data. The download site for CLUES is https://github.com/Wuchao1984/CLUESv1.

## Introduction

Mapping epigenomic modifications and chromatin regulator/transcription factor binding positions is critical for understanding human development and disease manifestation [1, 2]. Chromatin immunoprecipitation followed by high-throughput sequencing (ChIP-Seq) is widely used to map target proteins and modification positions on chromosomes quantitatively and at the genome-wide scale [3]. So far, individual researchers and consortium projects, such as ENCODE and Roadmap Epigenomics, have been generating an enormous amount of publicly available ChIP-Seq data [4, 5].

The field has developed many enriched regions (ERs) calling algorithms such as MACS2, SICER, MUSIC, PeakRanger, SISSRs, ZINBA, DFilter, HOMER, Hpeak, and QuEST, and each algorithm has its advantages [6–16]. MACS2 is widely used for calling narrow peaks, such as TFs binding sites. SICER is designed to identify broader ERs, such as H3K27me3 modification regions. MUSIC focuses on ERs with multiple length scales. In recent years, the discovery of broad enrichment signals such as broad H3K4me3 enrichment signals [17, 18], super-enhancer elements [1] and bivalent chromatin domains [19] pushes us to integrate multiple ChIP-Seq data to explore epigenetic regulatory mechanism and identify potential key developmental regulators. It becomes urgent to develop tools to discover and prioritize the broad enrichment signals from different ChIP-Seq data.

To this end, CLUES uses an adaptive-size-windows strategy to identify ERs and cluster them into broad enrichment signals. To evaluate the performance of CLUES, we compared it with MACS2, MUSIC, PeakRanger, SISSRs, and SICER on 227 ChIP-Seq datasets that included TFs (Ctcf and Nrsf) and HMs (H3K4me3, H3K27me3, and H3K36me3) from ENCODE project. CLUES is as accurate as MACS2 and better than other methods in prioritizing ERs with the motif of corresponding TFs. CLUES detected and clustered ERs into broad enrichment signals in 105 H3K4me3 datasets, 26 H3K27me3 datasets, and 34 H3K36me3 datasets. The results show CLUES performs better than other methods on revealing GO terms functions associated with the prioritized broad enrichment signals.

To further validate the value of CLUES, we prioritized broad enrichment signals of Nanog, Oct4, H3K4me3 and H3K27me3 with CLUES in mouse ES cell and identified novel regulators of mouse ES cell self-renewal and pluripotency with the help of a genome-wide CRISPR/Cas9 negative selection genetic screen. We successfully identified Fam60a, Zmynd8, and Abt1, which are either novel or have unconfirmed roles in mouse ES cells [20, 21] as novel regulators of mouse ES cell self-renewal and pluripotency.

## Results

### The CLUES algorithm

CLUES identifies ERs in each ChIP-Seq data and clusters them into short ERs cluster (SER) and long ERs cluster (LER) according to two measurements-fragment rate by distance (FR-D) and fragment rate by reads enrichment (FR-RE). SERs and LERs are broad enrichment signals identified by CLUES.

**Fig 1:**
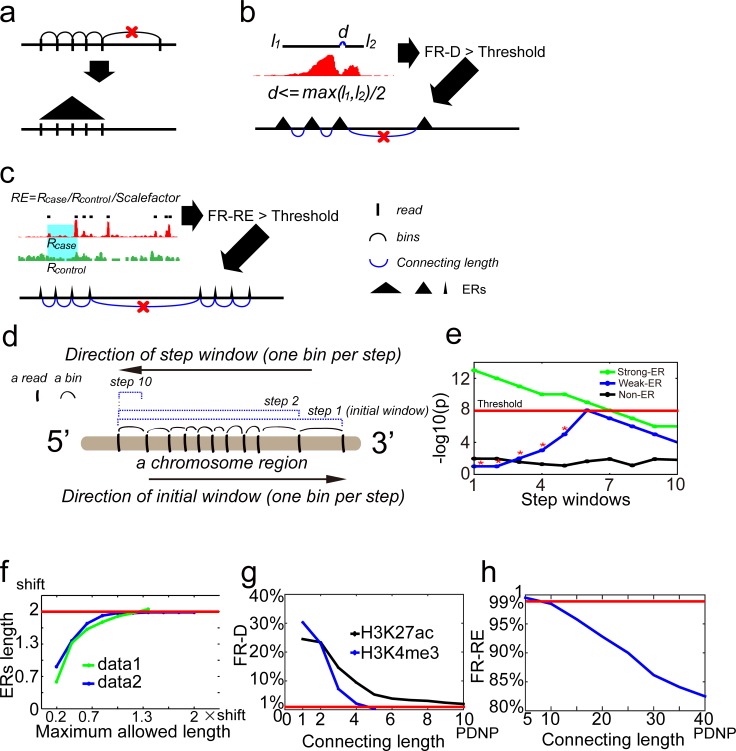
CLUES algorithm. a. CLUES clusters neighboring reads to be ERs. CLUES transforms distance between neighboring reads into a bin and merges bins smaller than maximum allowed length to be ERs. b. CLUES clusters neighboring ERs to be SERs. CLUES calculates fragment rate by distance (FR-D) of ERs in the dataset and clusters ERs to be SERs if the FR-D is significant. c. CLUES clusters neighboring ERs into LERs. CLUES calculates fragment rate by reads enrichment (FR-RE) of ERs in the dataset and clusters ERs to be LERs if the FR-RE is significant. d. A diagram showing how to build an initial window and the corresponding step windows. CLUES first sorts reads in the genome and converts two neighboring reads into a bin. Next, it combines 10 bins into an initial window. Then, CLUES shrinks the initial window into 10 step windows from 3’ to 5’ at one bin per step. For the next initial window, CLUES iterates the above two processes by moving one bin in the 5’ to 3’ direction. e. A plot of read enrichment of step windows in an initial window from simulated strong-, weak- and non-enriched regions. The step window with the highest enrichment is selected as the final window of an initial window. The X-axis corresponds to the serial number of step windows, and the Y-axis is the log p-value of read enrichment of the step windows. f. A plot depicts the ERs length under the different maximum allowed lengths from two Ctcf datasets. The X-axis is the maximum allowed length, and the Y-axis is the length of the 20th percentile of ERs length. g. A plot depicts the fragment rate by distance (FR-D) of SERs under the different connecting length of distance between neighboring peaks (PDNP; between 1th-10th PDNP) from H3K4me3 and H3K27ac datasets. h. A plot depicts the value of the fragment rate by reads enrichment (FR-RE) under the different connecting-length of distance between neighboring peaks (PDNP) from an H3K27me3 dataset.

CLUES links neighboring reads whose distance between them is smaller than a parameter named maximum allowed length to be ERs (Fig 1A). At first, CLUES transforms distance between two neighboring reads into a bin. Then, CLUES excludes the bins equal to or larger than maximum allowed length and links the remained continuous neighboring bins as an initial window, and it builds step windows inside the initial window (Fig 1D). Next, CLUES calculates read enrichment of the step windows by Poisson distribution test (see CLUES algorithm in Methods for details). At forth, CLUES selects the step window that has the highest read enrichment as final window of an initial window. At last, CLUES builds overlapping initial windows by moving a bin one step in genome and gets their final windows. It merges significant final windows (corrected *p* value < 0.05) to get ERs.

Through the step window strategy, CLUES successfully determines an appropriate final window to identify the weak ERs that are missed by larger windows (points with stars on the blue line in Fig 1E; here, we simulated the length of 10 neighboring bins to model a strong ER, a weak ER, and a non-enriched region.). To avoid calling oversized ERs, MACS2 estimates the size of most ERs as 2×shift and uses this information to set the length of windows to call ERs. CLUES extends this idea. CLUES uses a series of maximum allowed length parameters to adjust the length of initial windows to decrease oversized ERs (Fig 1F, CLUES algorithm in Methods for details). CLUES identifies the smallest maximum allowed length parameter which satisfies that 80% ERs’ length is larger than 2×shift and uses it to call ERs.

To call SERs, CLUES compares the distance between two neighboring ERs (*d*) with the ERs’ length (*l*_*1*_,*l*_*2*_) and counts the cases satisfying the formula in Fig 1B. CLUES enumerates the distances between every two neighboring ERs in genome and calculates the frequency of the cases satisfying the formula as FR-D. CLUES links ERs to be SERs with the connecting length parameter in the data with significant FR-D (default is 1%; users can set their threshold). It builds initial windows smaller than the connecting length parameter, identifies their final windows and merges the significant final windows to get merged windows. Then it identifies the shortest regions covering ERs in the merged windows as SERs. CLUES uses a series of connecting length parameters to adjust the length of initial windows and further the length of SERs. It calculates FR-D of the identified SERs under the different connecting length parameters (Fig 1G, see CLUES algorithm in Methods for details). Then it identifies the maximum connecting length parameter under which the SERs’ FR-D is smaller than the threshold (default is 1%, users can set their threshold) and reports these SERs.

To call LERs, at first, CLUES divides reads number in case sample by reads number in control sample to get *Scalefactor* parameter. Then CLUES counts reads in the shortest region covering two neighboring ERs in case (*R*_*case*_) and control (*R*_*control*_) samples and calculates reads enrichment of the region (*RE*) as the formula in Fig 1C. CLUES enumerates the shortest regions covering every two neighboring ERs in genome and calculates *REs* of the regions. CLUES calculates the frequency of the regions with *RE*>1.5 as FR-RE. CLUES links ERs to be LERs with the connecting length parameter of significant FR-RE (default is 1%; users can set their threshold). It builds initial windows smaller than the connecting length parameter and gets merged windows following the steps in SERs calling. Then it identifies the shortest regions covering the ERs in the merged windows as LERs. CLUES uses a series of connecting length parameters to adjust the length of initial windows and further the length of LERs. It calculates FR-RE of the ERs in LERs under the different maximum connecting length parameters (Fig 1H, see CLUES algorithm in Methods for details). CLUES identifies the maximum connecting length parameter which allows the LERs’ FR-RE is larger than the threshold (default is 99%, users can set their threshold) and reports these LERs. Replace the ERs described above with SERs, CLUES can link neighboring SERs to be LERs in the same way.

We described the details of CLUES algorithm in Methods section. We listed the parameters/thresholds of CLUES in S1 Table. Three thresholds, FR-D, FR-RE and RE, need be set to run CLUES. We have tested them in 227 ChIP-Seq data (S2 Table) and recommend default value for users. For the users who want to set their own FR-D/FR-RE threshold, we recommend them to get the value from the FR-D vs. connecting length plot of SERs and FR-RE vs. connecting length plot of LERs (Fig 1G and 1H). Users can select FR-D threshold as the value near the convergence point in FR-D vs. connecting length plot of SERs. Users can choose FR-RE threshold at the point where the curve tendency is changed in FR-RE vs. connecting length plot.

### CLUES is sensitive in identifying ERs from Ctcf and Nrsf ChIP-Seq data with high background noise

**Fig 2:**
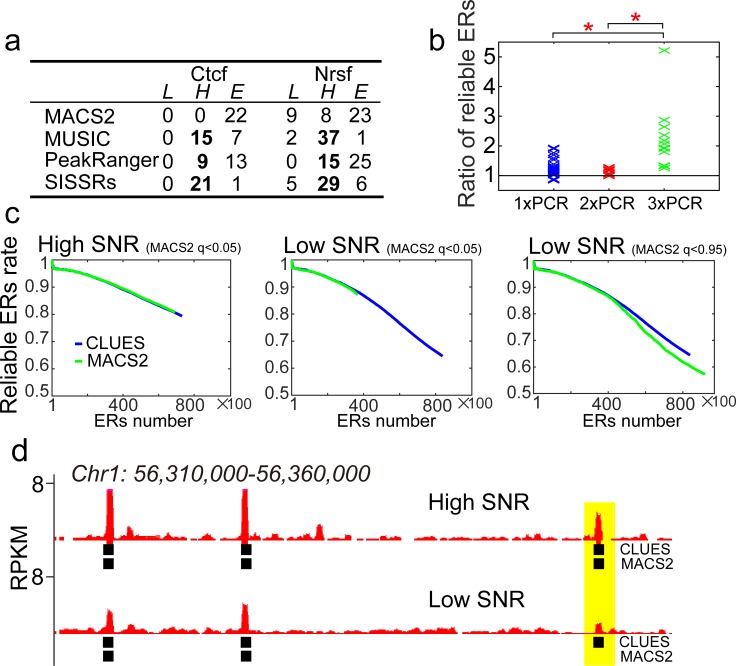
CLUES identifies ERs as accurate as MACS2 but detects more ERs. a. The comparison of the positive predictive value (PPV) between CLUES and rival methods (MACS2, MUSIC, PeakRanger, and SISSRs). "*L*", "*H*" and “*E*” show that CLUES has lower, higher and equal PPV than the rival method, respectively. The 22 Ctcf and 40 Nrsf datasets are used. Please find the description of "*L*", "*H*" and “*E*” in “Comparing the PPV of CLUES with the other methods in detecting reliable-ERs” part of Method section. b. The comparison of the reliable-ERs number between CLUES and MACS2 from ChIP-Seq datasets with different PCR amplification cycles. The Y-axis is the ratio of reliable-ER numbers detected by CLUES and MACS2 from a dataset. Regarding the labels, 1×PCR indicates approximately 10 amplification cycles. The 62 ENCODE Ctcf and Nrsf datasets are plotted. Asterisks (*) indicate that the difference of the ratio between the different PCR amplification groups is significant (Wilcoxon rank-sum test, p-value<0.0001). c. CLUES and MACS2 reliable-ERs-rate curves of two Ctcf datasets from a biological replicate pair with significantly different SNR. The Y-axis is the percentage of ERs with a motif(s), and the X-axis is the number of top-ranked ERs. Two q-values (0.05 and 0.95) were used for MACS2 to call ERs from the Low SNR dataset. d. The plots of Ctcf ERs identified by CLUES and MACS2 in a genomic region. The two Ctcf datasets with significantly different SNR from a biological replicate pair are used. The ERs with motifs detected by CLUES but not detected by MACS2 are highlighted. Y-axes, RPKM of Ctcf.

To assess the ability of CLUES to call ERs, we compared CLUES (ER calling module) with MACS2, MUSIC (TF-peaks mode), PeakRanger (ranger mode) and SISSRs under their default parameters using 22 Ctcf and 40 Nrsf ChIP-Seq datasets (S3 Table). CLUES identified more ERs from 54 datasets than all other methods except MUSIC. We considered ERs containing the known motif(s) ±150 bp from their summit as reliable ERs. To compare the positive predictive value (PPV) of ERs calling, we calculated the reliable-ERs-rate curves of the ERs identified by CLUES, MACS2, MUSIC, PeakRanger and SISSRs from the Ctcf and Nrsf ChIP-Seq datasets (see Methods for more details). We found that the performance of CLUES is the same as MACS2, but is better than MUSIC, PeakRanger, and SISSRs (Fig 2A; S4 Table). In the 62 ChIP-Seq datasets, 40 datasets were performed with 1×PCR, 10 datasets were performed with 2×PCR, and 12 datasets were performed with 3×PCR (1×PCR corresponds to approximately ten cycles of amplification). Increased PCR amplification can generate artificial enrichment within input controls, and this bias is a significant obstacle for ERs calling (S1 Fig). CLUES identifies more reliable ERs than MACS2 in 37 of 40 1×PCR datasets, 9 of 10 2×PCR datasets and all 3×PCR datasets at the default q-value (0.05 for both CLUES and MACS2). Considering that the reliable-ERs-rates are comparable for the equal number of ERs in these data, CLUES performs better than MACS2 in tolerating the PCR bias from input controls at a strict FDR level (Fig 2B; S2 Fig). Next, we studied the performance of MACS2 and CLUES with default parameters on the 20 1×PCR biological replicate pairs. We found that CLUES detects more reliable ERs than MACS2, especially on low-enrichment datasets (16 of 20 pairs) at a default q-value (0.05 for both CLUES and MACS2; Fig 2C and 2D and S3 Fig; see “Comparing the number of reliable-ERs identified by CLUES and MACS2 under different signal-to-noise (SNR) value” in Methods for more details). This indicates that CLUES also performs better than MACS2 in tolerating background noise in case samples at a strict FDR. When we relaxed the FDR of MACS2 (a q-value of 0.95) to increase the ERs number for the datasets from which MACS2 detects much fewer ERs than CLUES (MACS2 detects fewer than half the number of ERs detected by CLUES) at a strict FDR, we found that there are still fewer reliable ERs detected by MACS2 compared to CLUES (Fig 2C, S4 Fig). Thus, CLUES performs similarly to MACS2 regarding positive predictive value (PPV), but it performs better than MACS2 regarding sensitivity on low-quality datasets that have higher PCR amplification bias or background noise.

To assess MACS2 and CLUES on the detection of false positive ERs, we compared CLUES with MACS2 in three groups of samples. In the first group, we used one biological replicate of input samples as case and took another biological replicate of the input samples as control. Reads enrichment signals in both the case and control samples are weak (S5A Fig). The result shows that MACS2 detected no ERs, CLUES detected hundreds of ERs (S5A Fig). In the second group, we used one biological replicate of input samples which was over-amplified (30 cycles of PCR amplification) as case and took another biological replicate of the input samples which was normally amplified (10 cycles of PCR amplification) as control. Reads enrichment signals in case sample is much stronger than that in control sample (S5B Fig). The result shows that CLUES and MACS2 detected the similar number of ERs (S5B Fig). The ERs detected by MACS2 and CLUES are highly overlapped: 92.1% ERs detected by MACS2 are also revealed by CLUES. In the third group, we used the H3K27me3 ChIP-Seq data from mouse ES cell as case and took the input sample from mouse ES cell as control. Reads enrichment signals in case sample are stronger than that in control sample (S5C Fig), but the signals are weaker than that in the over-amplified sample of the second group (S5B Fig). We called ERs in the datasets by MACS2 and CLUES. CLUES detected much more ERs than MACSS2 (S5C Fig). Mouse Hoxa genes are targets of Polycomb proteins and form local 3D clusters centered on the H3K27me3 mark. The result shows that CLUES detected H3K27me3 signals enriched in Hoxa family and MACS2 failed to detect the H3K27me3 signals (S5D Fig). MACS2 failed to detect ERs from the dataset with weak reads enrichment signals. Taken three experiments together, suppose the true positive ERs are strong, we think MACS2 and CLUES should perform the same when false positive ERs are strong, and MACS2 would be a better choice when false positive ERs are weak. However, CLUES would be a better choice when the true positive ERs and false positive ERs are both weak in the data.

### CLUES prioritizes broad H3K4me3 enrichment signals implicating active cell functions

H3K4me3 HMs widely exist in the mammalian genomes. Recent studies show that broad H3K4me3 enrichment signals (broad H3K4me3 E-signals) play important roles in the epigenetic regulatory mechanism involved in development and disease processes[17, 18]. Prioritization of these broad H3K4me3 E-signals facilitates us to discover the key genes and cell functions being regulated by the epigenetic regulatory mechanism.

**Fig 3:**
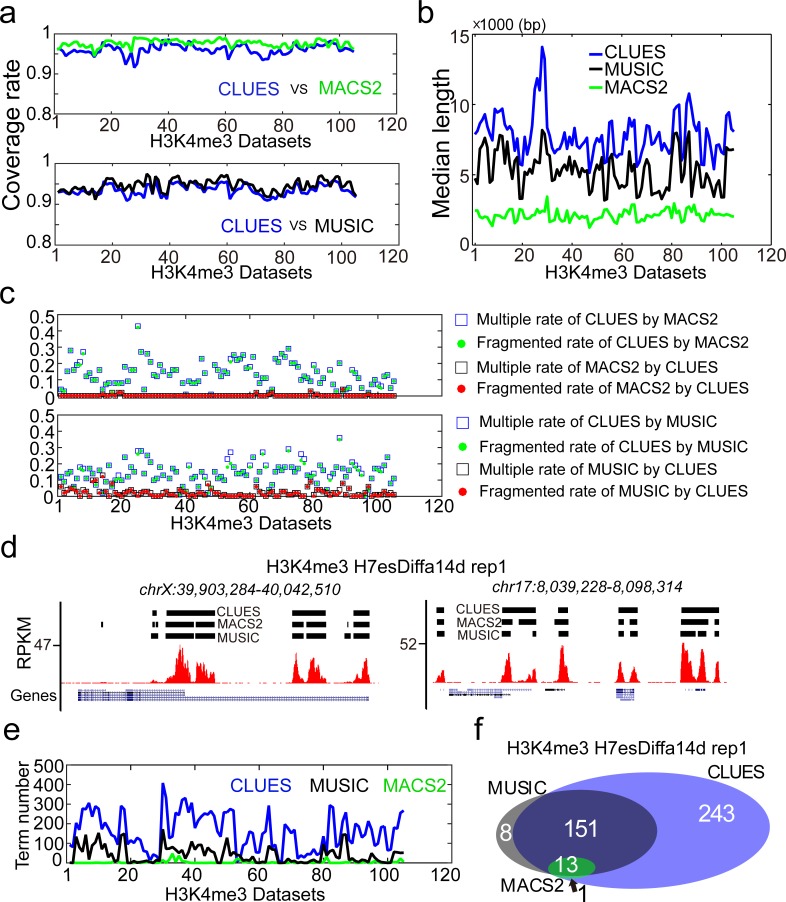
CLUES prioritizes broad H3K4me3 E-signals implicating active cell functions. a. The reciprocal coverage of the broad H3K4me3 E-signals identified by CLUES, MACS2, and MUSIC. The X-axis is the serial number of the 105 H3K4me3 datasets sorted by the first letter of their names from A to Z. The Y-axis is the percentage of a given method’s broad H3K4me3 E-signals revealed by its rival. b. The median length of top 100 broad H3K4me3 E-signals identified by CLUES, MACS2, and MUSIC from the 105 H3K4me3 datasets. c. Comparing the integrity of top 100 broad H3K4me3 E-signals identified by CLUES, MACS2, and MUSIC from the 105 H3K4me3 datasets. Multiple-rate is the percentage of a given method's top 100 broad H3K4me3 E-signals detected as multiple E-signals by its rival. The fragment rate is the percentage of the given method's top 100 broad H3K4me3 E-signals detected as fragmented E-signals by its rival. d. Plot broad H3K4me3 E-signals identified by CLUES, MACS2, and MUSIC from an H3K4me3 dataset at two genomic regions. e. The number of GO terms from top 100 broad H3K4me3 E-signals identified by CLUES, MUSIC, and MACS2 from the 105 H3K4me3 datasets. f. Venn-diagram of GO terms from top 100 broad H3K4me3 E-signals identified by CLUES, MUSIC, and MACS2 from an H3K4me3 dataset.

We used MUSIC (multiscale-punctate-ERs mode), MACS2 (broad-peak mode) and CLUES (SERs calling module) to detect broad H3K4me3 E-signals from 105 ChIP-Seq datasets with default parameters. The broad H3K4me3 E-signals identified by CLUES are highly overlapped with the broad H3K4me3 E-signals identified by MACS2 and MUSIC (Fig 3A). However, the prioritized broad H3K4me3 E-signals by the methods are different. The top-ranked broad H3K4me3 E-signals identified by CLUES are significantly longer than those top-ranked broad H3K4me3 E-signals identified by MACS2 and MUSIC (Fig 3B and S6 Fig). A significant proportion (>10%) of top 100 broad H3K4me3 E-signals identified by CLUES were identified as two or more separated broad H3K4me3 E-signals by MACS2 and MUSIC, whereas the top-ranked broad H3K4me3 E-signals identified by MACS2 and MUSIC were detected to be single ones by CLUES (Fig 3C and 3D, and S7 Fig). We associated top 100 broad H3K4me3 E-signals identified by the methods with their close genes in genome (see “GO analysis” in Methods for details) since H3K4me3 HMs are commonly viewed to be enriched in the promoter or enhancer regions of genes and influence genes expression. Around 100 genes were found to be associated with the top 100 broad H3K4me3 E-signals from CLUES, MACS2, and MUSIC in each H3K4me3 dataset (S5 Table). We found more GO terms from the associated genes of CLUES than from the associated genes of MUSIC and MACS2 (Fig 3E). The conclusion is the same for the associated genes of the top 1000 broad H3K4me3 E-signals from CLUES, MACS2, and MUSIC (S8 Fig). In some dataset, almost all GO terms from associated genes of top 100 broad H3K4me3 E-signals of MUSIC and MACS2 were covered by GO terms from associated genes of top 100 CLUES broad H3K4me3 E-signals (Fig 3F and S6 Table). We found that in 85% H3K4me3 datasets, more than 20% GO terms from associated genes of top 100 MUSIC broad H3K4me3 E-signals were overlapped with the GO terms from associated genes of top 100 CLUES broad H3K4me3 E-signals(S9A Fig, we excluded MACS2 result in this analysis because few GO terms were detected in most H3K4me3 datasets). We found in 94% H3K4me3 datasets, more than 80% GO terms from associated genes of MUSIC broad H3K4me3 E-signals were overlapped with the GO terms from associated genes of CLUES broad H3K4me3 E-signals when we kept the number of top-ranked MUSIC broad H3K4me3 E-signals as 100 and extended the number of top-ranked CLUES broad H3K4me3 E-signals as 1000 (S9B Fig). However, in 10% H3K4me3 datasets, more than 60% GO terms from associated genes from CLUES broad H3K4me3 E-signals were overlapped with the GO terms from associated genes of MUSIC broad H3K4me3 E-signals when we kept the number of top-ranked CLUES broad H3K4me3 E-signals as 100 and extended the number of top-ranked MUSIC broad H3K4me3 E-signals as 1000 (S9C Fig). This suggests if CLUES lowers the threshold of top-ranked broad H3K4me3 E-signals, its GO terms result will cover the GO terms result of MUSIC. CLUES has the ability to cover MUSIC on discovering GO terms from associated genes of prioritized broad H3K4me3 E-signals. On the contrary, MUSIC could not discover most GO terms of CLUES if MUSIC lowers the threshold of top-ranked broad H3K4me3 E-signals. CLUES can discover the GO terms missed by MUSIC. This GO term analysis shows the advantage of CLUES on revealing the active cell functions associated with broad H3K4me3 E-signals.

### CLUES prioritizes broad H3K27me3 enrichment signals implicating repressive cell functions

H3K27me3 HMs and H3K36me3 HMs are both epigenetic markers spreading up to 100kb in genome. Broad H3K27me3 E-signals reflect repressed chromatin structure and their abundance in gene body suggests repressed gene expression while broad H3K36me3 E-signals reflect open chromatin structure and are associated with activated gene expression[22]. Prioritization of broad H3K27me3 E-signals and H3K36me3 E-signals facilitates us to discover the active and repressive transcriptions and cell functions in the development and disease cells.

**Fig 4:**
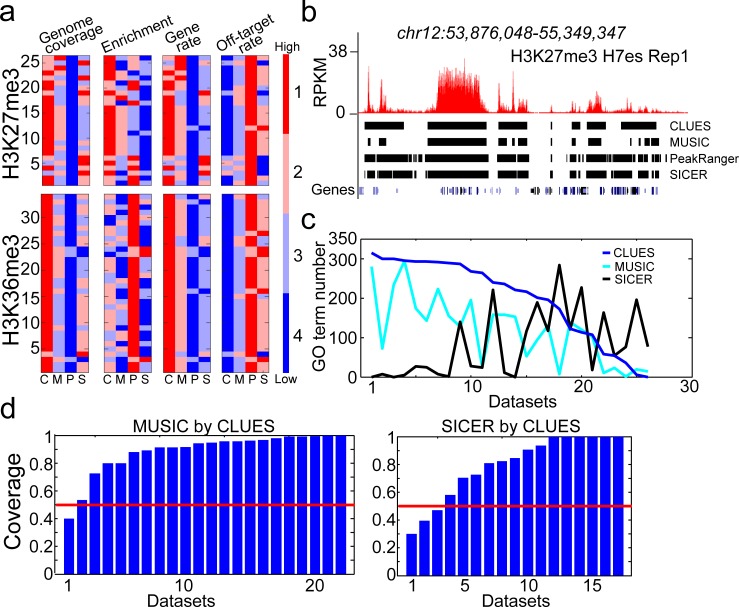
CLUES prioritizes broad H3K27me3 E-signals implicating repressive cell functions. a. The characteristics of top 100 broad E-signals identified by CLUES(C), MUSIC(M), PeakRanger(P) and SICER(S) from 26 H3K27me3 and 34 H3K36me3 datasets. The total length (Genome coverage), minimum reads-enrichment (Enrichment), the number of covered genes (Gene-rate) and the number of broad E-signals without genes (Off-target rate) are compared. Higher genome coverage, higher enrichment, higher gene-rate or lower off-target rate reflect a better performance of a method. The heat-maps are rank-ordered based on the first letter of dataset name from A to Z. b. The plot of H3K27me3 broad E-signals identified by CLUES, MUSIC, PeakRanger and SICER in a genomic region. Y-axis, RPKM of H3K27me3. c. The number of GO terms from top 100 broad E-signals identified by CLUES, MUSIC, and SICER from the 26 H3K27me3 datasets. The X-axis is the serial number of datasets. d. The coverage of GO terms from MUSIC and SICER broad E-signals by those from CLUES broad E-signals from 26 H3K27me3 datasets. Only the datasets with more than 10 GO terms in their top 100 broad E-signals identified by each method are used.

We compared broad E-signals identified by CLUES (LERs calling module) with those identified by MUSIC (multiscale-broad-ERs mode), PeakRanger (bcp mode), and SICER from 26 H3K27me3 and 34 H3K36me3 ChIP-Seq datasets using default parameters. The top-ranked broad E-signals of H3K27me3 and H3K36me3 prioritized by CLUES covered significantly wider genomic regions and more genes than the other methods without sacrificing the read fold-enrichment (Fig 4A and 4B, S10 Fig). Next, we associated top 100 broad H3K27me3 E-signals identified by the methods with their covering genes in genome (see “GO analysis” in Methods for details) to explore the repressed genes and cell functions by H3K27me3 HMs. As expected, a significant amount of GO terms can be found from the genes covered by top 100 CLUES, MUSIC and SICER broad H3K27me3 E-signals. Few GO terms were found from the genes covered by top 100 PeakRanger broad H3K27me3 E-signals, and we excluded PeakRanger on the following analysis. The GO terms from CLUES broad H3K27me3 E-signals are more than those from MUSIC broad H3K27me3 E-signals in 23 of 26 datasets and those from SICER broad H3K27me3 E-signals in 19 of 26 datasets (Fig 4C). Further analysis finds that the GO terms from CLUES broad H3K27me3 E-signals cover more than half of the GO terms from MUSIC broad H3K27me3 E-signals in 21 of 22 datasets and from SICER broad H3K27me3 E-signals in 14 of 17 datasets (Fig 4D). This analysis shows the advantage of CLUES on revealing the repressive cell functions associated with broad H3K27me3 E-signals.

### CLUES captures bivalent chromatin status and genes of core regulation circuitry of mouse ES cells from the integrative analysis of prioritized broad E-signals of Nanog, Oct4, H3K4me3 and H3K27me3

Mouse ES cells represent one of the best *in vitro* models of chromatin organization and epigenetic regulation [23, 24]. We used CLUES, MACS2, SICER and MUSIC to analyze H3K4me3, H3K27me3, Oct4 and Nanog ChIP-Seq data of mouse ES cells.

**Fig 5:**
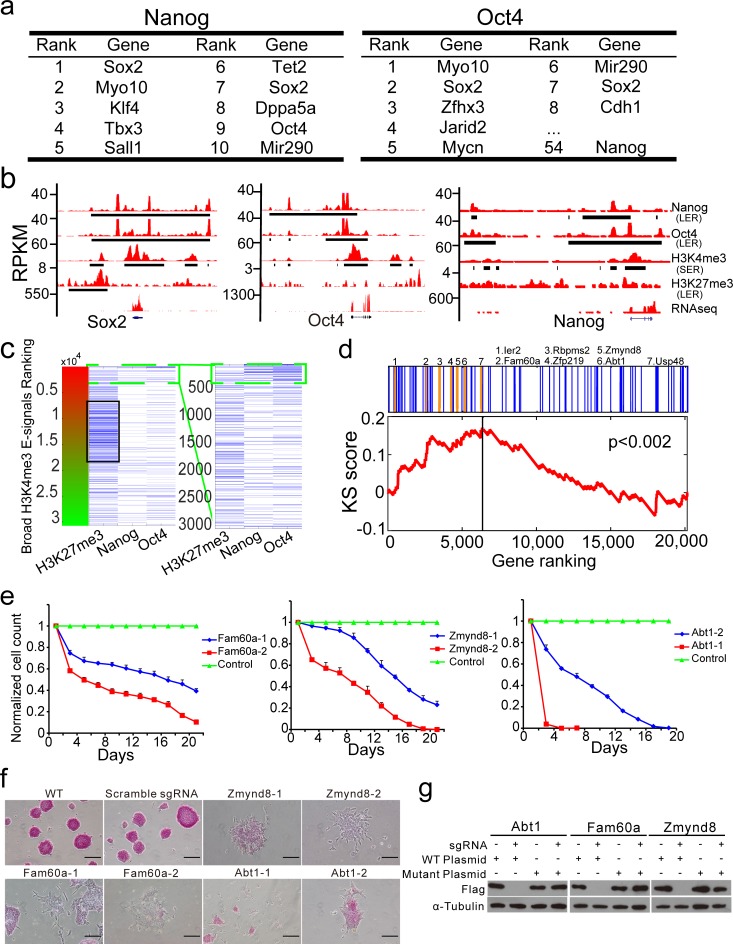
CLUES captures bivalent chromatin status, core regulation genes circuitry and novel self-renewal and pluripotency regulators of mouse ES cells by integrating prioritized broad E-signals of H3K4me3, H3K27me3, Nanog and Oct4. a. Genes associated with top-ranked broad E-signals of Nanog and Oct4. b. The plots of broad E-signals of H3K4me3, H3K27me3, Nanog and Oct4 and RNA-Seq signals at Sox2, Oct4 and Nanog locus. Y-axes, RPKM of Nanog, Oct4, H3K4me3, and H3K27me3 ChIP-Seq datasets and RNA-Seq datasets. c. A heat-map of broad H3K4me3 E-signals associated with broad H3K27me3 E-signals, top-ranked Nanog (top 5%) and top-ranked Oct4 (top 5%) broad E-signals. The heat-map is rank-ordered by broad H3K4me3 E-signals. d. The top 100 genes revealed by the CLUES integrated analysis are significantly enriched at the top of the list from a CRISPR/Cas9 negative selection genetic screen (Kolmogorov–Smirnov test, p<0.002). Genes in the list are ranked by their screen scores (see Methods for more details). The seven highlighted genes were subjected to further experimental verification for their roles in mouse ES cells. e. Knockout of Fam60a, Zmynd8 or Abt1 significantly decreases the proliferation of mouse ES cells. Each gene is targeted by three different CRISPR-sgRNAs, and the effective ones are shown. The graph plots the percentages of mutant ES cells normalized against wild-type ES cells. Error bars indicate the SD of triplicates. f. Knockout of Zmynd8, Fam60a or Abt1 significantly increases spontaneous differentiation of mouse ES cells, as indicated by loss of the pluripotency marker alkaline phosphatase (AP) and flattened colonies.g. Western blotting shows that silent mutation of Abt1, Fam60a, and Zmynd8 resistant to corresponding CRISPR-sgRNA targeting *in vivo*. Controls, wild-type genes. Loading controls, tubulin.

We identified broad H3K4me3 E-signals with CLUES, MACS2, SICER, and MUSIC, setting the species parameter as "mouse" and running the methods with the commands and parameters listed in S3 Table. GO terms of genes marked by top 100 broad H3K4me3 E-signals prioritized by CLUES, MACS2, SICER and MUSIC were analyzed. The results showed that CLUES revealed more GO terms than the other methods, and the GO terms are mostly related to fundamental biological processes and stem cell maintenance (S7 and S8 Tables). We identified broad E-signals of Nanog and Oct4 with CLUES (see “Calling Nanog LERs and Oct4 LERs” in Methods for detail). We also identified broad E-signals of Nanog and Oct4 with SICER and MUSIC (see S3 Table for the detailed commands and parameters, except for the species parameter, which was set as "mouse"). Per the results from CLUES, as expected, top-ranked broad E-signals of Nanog and Oct4 were mostly associated with key regulators of pluripotency and self-renewal of mouse ES cells (Fig 5A). The idea that Oct4, Sox2, and Nanog are regulated by both broad E-signals of Nanog and Oct4 is consistent with the previous reports that Oct4, Sox2, and Nanog collaborate to form regulatory circuitry consisting of auto-regulatory and feed-forward loops [25, 26] (Fig 5B). The GO terms of genes (genes located in 1kb around broad E-signals) marked by top 100 broad E-signals of Nanog and Oct4 prioritized by SICER and MUSIC were analyzed. Fewer GO terms were revealed by the two methods, but the GO terms were also associated with pluripotency and self-renewal of mouse ES cells (S7 and S8 Tables).

Next, we integrated the information from broad E-signals of H3K4me3, H3K27me3, Nanog and Oct4 prioritized by CLUES. It is known that H3K27me3, together with H3K4me3, marks chromatin in a bivalent state, which is a poised condition of transcriptional activation in mouse ES cells [19, 27, 28]. Consistently, broad H3K27me3 E-signals (called by LERs calling module) tended to associate with broad H3K4me3 E-signals, which had a moderate ranking, whereas top-ranked broad E-signals of Oct4 and Nanog were associated with highly ranked broad H3K4me3 E-signals (Fig 5C; see “Integrating H3K4me3 SERs, H3K27me3 LERs, Oct4 LERs and Nanog LERs in mouse ES cells” in Methods for more details). To search novel genes that may be essential for the pluripotency and self-renewal of mouse ES cells, we selected genes with specific epigenetic markers structure: the genes should be close to or overlapped with top-ranked broad Nanog/Oct4 E-signals and top-ranked broad H3K4me3 E-signals in the genome and should not be covered by broad H3K27me3 E-signals. This epigenetic markers structure is similar to super-enhancers composing by broad transcription factor E-signals and broad active histone modifications E-signals. We believe the genes with this specific epigenetic markers structure could be promising candidate regulatory genes of cell identity. We identified genes with this specific epigenetic markers structure in the prioritized broad E-signals of Nanog/Oct4, H3K4me3, and H3K27me3 of CLUES, MUSIC, and SICER. We filtered candidate genes with the following criteria: 1.the gene is located 1kb around or in top 300 broad H3K4me3 E-signals; 2. the gene is located 10kb around or in top 1000 broad E-signals of Nanog/Oct4; 3. the gene is not covered by any broad H3K27me3 E-signals. Among 100 identified genes, 39 were well-known regulators such as Esrrb [1], Utf1 [29], Tet1 [30], Oct4 [31] and Sox2 [31], and 61 genes were potential novel genes that needed to be further investigated for their roles in mouse ES cells (S9 Table). We also filtered genes with the criteria for the top-ranked broad E-signals of H3K4me3, H3K27me3, Oct4 and Nanog prioritized by SICER and MUSIC. Ten genes were identified from the SICER results, which represented 10% of the identified genes from the CLUES results. Forty-four genes were identified from the MUSIC results. Among the 44 genes, 21 of them are also revealed from CLUES’ result (S9 Table).

### CLUES facilitates confirming regulators of self-renewal and pluripotency of mouse ES cells from a CRISPR/Cas9 negative selection genetic screen

Genes regulated by Oct4 and Nanog LERs may function in self-renewal and pluripotency of mouse ES cells [32, 33]. We performed a genome-wide CRISPR/Cas9 negative selection genetic screen on mouse ES cells. A total of 20,801 genes were targeted by 61,804 sgRNAs at a multiplicity of infection (MOI) of approximately 0.3 [34]. We used each sgRNA as a barcode to count its host cells at the beginning of the experiment and 20 days after transduction (the 10th generation), and genes that may positively contribute to mouse ES cell proliferation were prioritized (see “Prioritizing genes that contributed to ES cells proliferation from a genomic-wide negative selection genetic screen” in Methods for more detail). As expected, many well-known regulators were top-ranked, such as Nanog (ranked 241th), Oct4 (ranked 551th) and Utf1 (ranked 691th). However, further GO term analysis showed that top-ranked genes (690 genes ahead of Utf1) are mostly related to fundamental processes (S11 Fig), so it was not easy to confirm novel regulators of mouse ES cell self-renewal and pluripotency directly. Through the Kolmogorov–Smirnov test [35], we found that 10 genes shortlisted by SICER and 44 genes shortlisted by MUSIC showed no significant enrichment at the top of the gene prioritization list of the CRISPR-sgRNA screen (SICER: P<0.703, MUSIC: P<0.293, S12 Fig), but 46 genes of the 100 genes shortlisted by CLUES were significantly enriched at the top of the gene prioritization list of the CRISPR-sgRNA screen (P<0.002), and seven genes among these had either unknown or not fully confirmed roles in mouse ES cells [20, 21] (Fig 5D). This result encouraged us to knock out these genes individually in mouse ES cells to confirm their functions. We finally confirmed that mouse ES cells lacking Zmynd8 [20, 36], Abt1 [37] or Fam60a [21, 38] showed a decrease in proliferation rate and an increase in differentiation (Fig 5E, S13 Fig). Additionally, the slow proliferation of mutated mouse ES cells could be partially reversed by transient re-expression of a silent mutation of the corresponding gene which resists gRNA targeting (Fig 5F and 5G, S14–S16 Figs). Thus, CLUES helped show that Zmynd8, Abt1, and Fam60a are novel regulators of the renewal and pluripotency of mouse ES cells based on the CRISPR/Cas9 negative selection genetic screen.

CLUES helped narrow the gene list of CRISPR/Cas9 negative selection genetic screen to 46 candidate genes. Three of them, Zmynd8, Abt1, and Fam60a were confirmed to be novel regulators of self-renewal and pluripotency of mouse ES cells. We also narrowed the above gene list by SICER and MUSIC to include Zmynd8, Abt1, and Fam60a in the candidate gene set. Finally, we obtained 4118 candidate genes for SICER and 508 candidate genes for MUSIC (see “Narrow down gene prioritization list of the genomic-wide negative selection genetic screen by SICER and MUSIC” in Methods for more details). The number of candidate genes showed that CLUES effectively helped confirm three novel regulators from the CRISPR/Cas9 negative selection genetic screen compared to SICER and MUSIC.

## Discussion

We present CLUES, a novel algorithm for identifying and prioritizing broad enrichment signals from different types of ChIP-Seq. CLUES first detects ERs, and then clusters them to be SERs and LERs. An ER corresponds to the individual binding site of a transcription factor (TF) or a histone modification (HM). An SER corresponds to continuous binding sites of a TF or an HM. The binding sites are close in the genome. An LER corresponds to discontinuous binding sites of a TF or an HM. The binding sites frequently appear in a genome region, but they may be not close enough. In a TF ChIP-Seq data, an SER suggests a DNA element bound by the TF and the TF may be a self-interaction protein; an LER suggests a super-enhancer bound by the TF. In an HM ChIP-Seq data, an SER suggests a promoter/enhancer/super-enhancer bound by the HM; an LER suggests an active/repressive chromatin domain bound by the HM. CLUES, tested on 227 ChIP-Seq datasets from the ENCODE project, showed significant advantages on prioritizing broad enrichment signals implicating active/repressive cell functions associated with the epigenetic regulation mechanism. This characteristic is convenient for the integrated analysis of H3K4me3, H3K27me3, Nanog and Oct4 ChIP-Seq data of mouse ES cells and helped us re-capture the bivalent chromatin stage and the core regulation circuitry in mouse ES cells.

Genetic screens play an important role in exploring regulators of a given phenotype [39]. These methods have led to the discovery of the roles of genes or entire pathways in various development or disease processes. The recently developed CRISPR/Cas9 system is a robust genetic screen approach that has helped in identifying many disease targets for novel therapies, mostly from positive selection screens designed to screen inhibitors of a biological process [40, 41]. However, it is still difficult to confirm essential genes of a biological/disease process from a negative selection genetic screen because of the overwhelming noise from housekeeping genes [42]. Cell-type specific chromatin structures, including transcription factor binding and histone modifications, maintain the expression of genes that define cell identity [33]. In this paper, we used CLUES to capture the specific chromatin structures of mouse ES cells and their regulating genes. The shortlisted genes helped to avoid the interference of housekeeping genes and confirmed novel regulators of mouse ES cell self-renewal and pluripotency from a negative selection genetic screen.

## Conclusions

Here we present a novel algorithm, CLUES, to identify and prioritize broad E-signals from different types of ChIP-Seq data. CLUES is a promising tool for the integrative analysis of broad enrichment signals from different types of ChIP-Seq data. The confirmation of 3 novel regulators of mouse ES cell self-renewal and pluripotency from a CRISPR/Cas9 experiment not only shows the power of CLUES for integrated analysis of ChIP-Seq data but also shines a light on a hybrid computational-experimental strategy for screening essential genes of a biological/disease process.

## Methods

### Data and data processing

We downloaded the ChIP-Seq datasets of Ctcf, Nrsf, H3K4me3, H3K27me3, and H3K36me3 of human cells from the ENCODE project to call ERs and broad E-signals. We downloaded the ChIP-Seq datasets of Nanog, Oct4, H3K4me3, and H3K27me3 of mouse ES cells to identify novel regulators of ES cell self-renewal and pluripotency. The ChIP-Seq data were mapped to the reference genome of hg19 and mm9 using bowtie accordingly [43]. Two mismatches were allowed, and only uniquely mapped reads were reported during the mapping process. Then, duplicate reads were filtered using Samtools [44]. The detailed dataset information is listed in the S2 Table.

### CLUES algorithm

CLUES accepts mapped reads as input and outputs ERs, SERs, and LERs. CLUES includes one reads-shifting module and three enriched-region calling modules (ERs Calling, SERs Calling, and LERs Calling). SERs and LERs are broad E-signals identified by CLUES. Here, we describe it in detail.

#### Read shifting

For single-end ChIP-Sequencing data, CLUES generates a series of shift parameters from zero to the value inputted by the user (DNA fragment size of ChIP-Seq library).

1. CLUES first shifts mapped reads towards their 3' end by the first shift parameters (the smallest one) and uses the 5' positions of shifted reads to sort the reads in the genome. The distance between two neighboring reads is transformed into a bin (S17A Fig).
2. CLUES calculates the size distribution of all bins and employs a bin-length vs bin-number plot (S17B Fig) to identify the short bins, which are the bins on the left side of the peak (or the first peak) in the plot. It records the proportion of short bins.
3. CLUES iterates the above two steps using different shift parameters until CLUES gets the highest proportion of short bins (S17B Fig). Thus, CLUES acquires an optimized-shift parameter.
4. CLUES shifts all mapped reads with the optimized-shift-parameter and then converts them into bins.

The test on 22 Ctcf ChIP-Seq datasets shows the shift parameters estimated by CLUES are highly consistent with the ones of MACS2 (S17C Fig).

For paired-end ChIP-Seq data, CLUES does not shift mapped reads. Instead, CLUES uses the middle-point of a set of paired reads to build bins directly, and it estimates the optimized-shift-parameter as half of the median size of all paired-end reads.

#### ERs calling

1. CLUES generates a series of maximum-allowed-length parameters from 0.2×optimized-shift-parameter to 2×optimized-shift-parameter. The first maximum-allowed-length parameter is 0.2×optimized-shift-parameter.
2. CLUES extracts reads from Chr1 to Chr5 in a ChIP-Seq data as the training set. It builds initial windows that include 20 bins from 5' to 3' until the last bin of a chromosome with one bin per step.
3. CLUES shrinks the initial window into step windows with one bin per step. Step windows with bins larger than or equal to the maximum-allowed-length parameter are excluded.
4. CLUES employs the Poisson model to calculate read enrichment in the step windows and calculates the final window from the step windows. It involves the following steps:
  (i). Count reads in a step window in a case sample (*R*_*window*_*case*_) and the corresponding region in the control sample (*R*_*window*_*control*_).
  (ii). Calculate reads background of the step window (*R*_*window*_*background*_) as
    $$R_{window\_background}={max(d}_{genome}\times l_{window},R_{window\_control}\times SF),$$
    $$d_{genome}=R_{case}/(l_{genome}\times MCF),$$
    $$SF=R_{case}/R_{control},$$
    where *d*_*genome*_ is the average read density of a case sample; *l*_*window*_ is the length of a step window; *SF* is the scale factor of the number of mapped reads in case sample (*R*_*case*_) and the number of mapped reads in control sample (*R*_*control*_); *l*_*genome*_ is the length of the reference genome, and *MCF* is the correction factor to correct the mappability of repeat regions in a genome [45].
  (iii). Calculate p-value of reads enrichment in the step window by Poisson distribution function in R as
    $$p=ppois((R_{window\_case}-1),R_{window\_background},lower=FALSE).$$
  (iv). Select the step window with the most significant *p*-value as the final window.
5. CLUES merges reads-enriched final windows (corrected *p*-value<0.05) to get merged windows set and calculates the length distribution of all merged windows in the training set.
6. CLUES iterates steps 2–5 using different maximum-allowed-length parameters until 80% of the merged windows are longer than 2×optimized-shift-parameter or the maximum-allowed-length parameter reaches 2×optimized-shift-parameter, whichever comes first (Fig 1F). Thus, CLUES acquires the optimized maximum-allowed-length.
7. CLUES calls ERs with the optimized-maximum-allowed-length.

#### SERs calling

1. CLUES calculates the distances between two neighboring ERs. It regards two ERs as fragmented ERs if the distance is less than half of the length of either ERs (Fig 1B).
2. CLUES calculates the fragment rate by distance (FR-D) of ERs in a ChIP-Seq data. If FR-D>0.01 (default value, users can set their threshold), CLUES will call SERs.
3. CLUES shuffles ERs in the genome to get simulated ERs. It calculates the distances between neighboring simulated ERs, ranks them from small to large and records the distances located at the 1st to 10th percentile of the rank list as the values of the 1st to 10th percentile of the distance between neighboring peaks (PDNP). CLUES keeps the PDNP parameters that are smaller than 10 kb.
4. CLUES extracts reads from Chr1 to Chr5 as the training set. It builds initial windows that include *x* bins from 5' to 3' until the last bin of Chr1 with one bin per step. *x* is calculated as follows:
  $$x=min(max(floor(l_{PDNP}\times d_{genome}\times 5),20),500),$$
  where *d*_*genome*_ is the average read density of case sample. *l*_PDNP_ is the value of a selected PDNP parameter, which starts from the first PDNP.
5. CLUES shrinks the initial window into step windows with one bin per step. The step windows larger than or equal to the *l*_PDNP_ are excluded.
6. CLUES employs the Poisson model to calculate the read enrichment of the step windows and obtains a final window from the step windows the same way as ERs calling.
7. CLUES merges reads-enriched final windows (corrected *p*-value<0.05) into merged windows.
8. CLUES trims and filters the merged windows by ERs: for a merged window overlapping a single ER, CLUES keeps the ER as the merged window; for a merged window covering multiple ERs, CLUES keeps the shortest region covering all ERs as the merged window; CLUES discards the merged windows covering no ERs. CLUES calculates FR-D of the merged windows.
9. CLUES iterates steps 3–8 using *l*_PDNP_ with different PDNP parameters (1th to 10th PDNP) until FR-D reaches 1% or the FR-D reaches a minimum, whichever comes first (Fig 1G). Thus, CLUES acquires the optimized *l*_PDNP_.
10. CLUES repeats steps 3–8 with the optimized *l*_PDNP_ to call merged windows as SERs.

#### LERs calling

CLUES clusters input enrichment signals (either ERs or SERs) to be LERs. Here, we cluster ERs as an example.

1. CLUES calculates fold-change of read enrichment of the shortest region covering two neighboring ERs in the ChIP-Seq data. The fold-change value (*RE*) is calculated as Fig 1C shows. CLUES regards the neighboring ERs as fragmented ERs if their *RE* is larger than 1.5. CLUES calculates FR-RE of ERs in the data. If FR-RE>0.01, CLUES calls LERs in the data.
2. CLUES shuffles ERs in the genome to get simulated ERs. It calculates the distances between neighboring simulated ERs, ranks them from small to large and records the distances as the values of PDNP for every 5 percentile from the 5th to 50th percentile. CLUES keeps PDNP parameters that are smaller than 100 kb.
3. CLUES repeats steps 2–5 of *ERs calling* to call merged windows in the training set, starting *l*_PDNP_ with the 5th PDNP.
4. CLUES trims and filters the merged windows by ERs: for a merged window overlapping a single ER, CLUES keeps the ER as the merged window; for a merged window covering multiple ERs, CLUES keeps the shortest region covering all ERs as a merged window. CLUES calculates the FR-RE of ERs in the merged windows.
5. CLUES iterates steps 3–4 above using *l*_PDNP_ with different PDNP parameters until the FR-RE decreases to 99% or *l*_PDNP_ reaches a maximum, whichever comes first (Fig 1H). Thus, CLUES acquires an optimized *l*_PDNP_.
6. CLUES runs the above steps 3–4 with the optimized *l*_PDNP_ to call merged windows as LERs.

Replace ERs with SERs, CLUES uses the same way to call LERs from SERs.

#### Reporting ERs, SERs, and LERs

CLUES reports length, reads number, p-value, q-value and fold-change value for enrichment signals of ERs, SERs, and LERs, and reports summits for ERs as follows:

1. CLUES calculates the length of an enrichment signal as *l*_*ER*_.
2. CLUES counts the read number of the enrichment signal in the case-sample as *R*_*ER*_*case*_.
3. CLUES calculates the p-value of the enrichment signal as follows:
  (i). Calculate the read background of the enrichment signal (*R*_*ER*_*background*_) as
    $$R_{ER\_background}={max(d}_{genome}\times l_{ER},R_{ER\_control}\times SF),$$
    here,
    $$d_{genome}=R_{case}/(l_{genome}\times MCF),$$
    $$R_{ER\_control}=d_{local\_control}\times l_{ER},$$
    $$d_{local\_control}=\left\{ \begin{array}{l} \max\left(d_{ER},d_{1kb},d_{10kb}\right),\;l_{ER}\leq 1kb\\ \max\left(d_{ER},d_{10kb}\right),\;{1kb<l}_{ER}\leq 10kb\\ d_{ER},\;l_{ER}>10kb \end{array},\right.$$
    $$SF=R_{case}/R_{control},$$
    where *d*_*genome*_ is the average read density of the case sample; *d*_*ER*_ is the read density of the corresponding region of the enrichment signal in control sample; *d*_1*kb*_ and *d*_10*kb*_ are the read density in the corresponding 1 kb and 10 kb regions around the enrichment signal in the control sample; *l*_*genome*_ is the length of the reference genome; *MCF* is the correction factor to correct the mappability of repeat regions in a genome[45]; *SF* is the scale factor of the number of mapped reads in the case sample (*R*_*case*_) and the number of mapped reads in the control sample (*R*_*control*_).
  (ii). Calculate the p-value of the read enrichment of the enrichment signal by a Poisson distribution function in R language as:
    $$p=ppois((R_{ER\_case}-1),R_{ER\_background},lower=FALSE).$$
4. CLUES calculates the q-value from the p-value of the called enrichment signals by Benjamini & Yekutieli method [46].
5. CLUES calculates the fold-change (*FC*_*ER*_) value of the called enrichment signals as:
  $${FC}_{ER}=R_{ER\_case}/R_{ER\_background},$$
  where $R_{ER_{case}}$ and *R*_*ER*_*background*_ are calculated in the same way as CLUES did in the third step.
6. CLUES calculates the summit of an ER as follows: each read position is extended optimized-shift-parameter bases from its center for the reads in the ER; the location with the highest fragment pileup is referred to as ‘the summit’.

#### Ranking of ERs and broad E-signals identified by the methods

The commands for calling ERs and broad E-signals by the methods are summarized in S3 Table.

We used a q-value of 0.05 to run CLUES and a default q-value (0.05) to run MACS2, a default q-value (0.001) to run MUSIC and a default FDR (0.0001) to run PeakRanger. Because there was no adjusted P-value option available, we used a default P-value (0.001) to run SISSRs. We ranked ERs identified by MACS2, MUSIC, PeakRanger by their adjusted P-value in ascending order and the ERs identified by SISSRs by their P-value in ascending order (for the ERs with equal adjusted-P-value or P-values, we ranked them by their read fold-change value in descending order). To rank the ERs identified by CLUES, we first ranked their q-value in ascending order and recorded the rank of each ER as *Rank*_*ER*_*qvalue*_; then, we ranked the ERs again by the summit height of each ER in descending order and recorded the rank of each ER as *Rank*_*ER*_*summit*_; finally, we ranked the ERs by the sum value of *Rank*_*ER*_*qvalue*_ and *Rank*_*ER*_*summit*_ of every ER in ascending order.

To identify broad H3K4me3 E-signals, we set the q-value to 0.05 to run CLUES, then we used a default q-value (0.1) to run MACS2 and a default q-value (0.001) to run MUSIC. We ranked the broad H3K4me3 E-signals identified by MACS2 and MUSIC by their q-value in ascending order (for broad H3K4me3 E-signals with equal q-values, we ranked them by their read fold-change value in descending order). We ranked the broad H3K4me3 E-signals identified by CLUES by their read number in ascending order.

To identify broad E-signals of H3K27me3 and H3K36me3 by CLUES, PeakRanger, SICER, and MUSIC, we set the q-value at 0.05 to run CLUES, the FDR at 0.05 to run SICER, a default q-value (0.001) to run MUSIC and a default FDR (0.0001) to run PeakRanger. We ranked the broad E-signals identified by SICER, MUSIC, and PeakRanger by their adjusted P-value in ascending order (for the broad E-signals with equal adjusted P-value, we ranked them by their read fold-change value in descending order). To rank the broad E-signals identified by CLUES we first ranked their fold-change value in descending order and recorded the rank of each broad E-signal as *Rank*_*LER*_*enrichment*_; then we ranked the broad E-signals by their length in descending order and recorded the rank of each broad E-signals as *Rank*_*LER*_*length*_; finally, we ranked broad E-signals by the sum value of *Rank*_*LER*_*enrichment*_ and *Rank*_*LER*_*length*_ of every broad E-signal in ascending order.

#### Comparing the PPV of CLUES with the other methods in detecting reliable-ERs

We defined ERs with the corresponding Ctcf or Nrsf motifs within 150 bp around their summits as reliable-ERs [8]. We compared the PPV of CLUES to the other methods in detecting reliable-ERs in the ChIP-Seq data of Ctcf and Nrsf.

We conducted the comparison using the following steps:

1. We identified ERs with the various methods and ranked the ERs as described in “Ranking of ERs and broad E-signals identified by the methods”.
2. We downloaded Ctcf and Nrsf motifs from the MEME database [47] (S10 Table) and scanned the corresponding motifs 150 bp upstream and downstream of the summits of the ERs by FIMO with a default p-value (0.0001) [48].
3. We calculated reliable-ERs rated in the top 100, 200, and 300 …, ERs of each method and fitted their reliable-ERs-rate curves.
4. We calculated the areas bounded by the X axis, Y axis and the reliable-ERs-rate curves of the same number of top-ranked ERs identified by CLUES and the other methods being compared.
5. We divided the area of a rival method (MACS2, MUSIC, PeakRanger or SISSRs) by the area of CLUES. The *Ratio* > 1.01 indicates CLUES has lower PPV than the rival method ("*L*" in Fig 2A); *Ratio* < 0.99 indicates CLUES has higher PPV than the rival method ("*H*" in Fig 2A); 0.99≤ *Ratio* ≤ 1.01 indicates CLUES has equal PPV with the rival method ("*E*" in Fig 2A). The ratios of the rival methods against CLUES for 62 Ctcf and Nrsf ChIP-Seq data are summarized in S4 Table.

#### Comparing the number of reliable-ERs identified by CLUES and MACS2 under different signal-to-noise (SNR) values

In each ChIP-Seq data, CLUES shifts reads according to the estimated shift parameter, then it calculates the size distribution of all bins and employs a bin-length vs. bin-number plot (S17B Fig) to identify the short bins, which are the bins on the left side of the peak (or the first peak) in the plot. It records the proportion of short bins. We used the proportion of short bins to assess the signal-to-noise ratio (SNR) of ChIP-Seq datasets at the same mapping depth. We calculated SNR of Ctcf and Nrsf ChIP-Seq data as follows:

1. We sampled 5 million reads from each data, then we calculated the proportion of small bins in the data as the value of SNR.
2. We calculated the ratio (*Ratio*_*C*/*M*_) of the number of reliable-ERs identified by CLUES and MACS2 under default q<0.05 in each data set.
3. We associated the SNRs with *Ratio*_*C*/*M*_ between biological replicate datasets.

#### GO analysis

At first, we associated top 100 and 1000 SERs and LERs in each data with genes as follows:

1. We downloaded gene location information from UCSC Genome Browser (hg19 version and mm9 version) [49].
2. A gene overlapped by or located within 1 kb of an SER was recognized as an SER-associated gene. A gene with 80% of its length covered by an LER was recognized as an LER-associated gene.

Then, we took the SERs-associated genes or LERs-associated genes as target set. We took all human (or mouse) genes as the background set. We employed the hypergeometric distribution test as enrichment function. We used GOstats to conduct GO analysis on the associated genes [50]. The database was from org.Hs.eg.db and org.Mm.Hs.eg.db, and the test method was the GOHyperGParams method. GO terms with p<10^−5^ are reported.

#### Calling Nanog LERs and Oct4 LERs in mouse ER cells

It is known that the pluripotency and self-renewal of mouse ES cells are potentiated by core TFs Oct4, Nanog and Sox2, and their binding sites frequently cluster in close genomic proximity to activate the expression of downstream genes [25, 51–53]. Because the H3K4me3 modification is required for activating expression [54, 55], the binding clusters of Oct4 and Nanog should couple with H3K4me3 SERs. Therefore, we used the LERs calling module to learn the connecting length (*l*_PDNP_) that allows the LERs of Nanog and Oct4 to have the most similar length compared to H3K4me3 SERs

1. We called ERs and SERs in H3K4me3 ChIP-Seq data by CLUES.
2. We called ERs and SERs in Nanog ChIP-Seq data by CLUES.
3. We called Nanog LERs from Nanog SERs as following:
  (i). We conducted steps 2–4 in “*LERs calling*” of the CLUES algorithm.
  (ii). We iterated steps 2–4 in “*LERs calling*” using *l*_PDNP_ with different PDNP parameters and recorded the *l*_PDNP_ where FR-RE > 99%.
  (iii). We called different Nanog LERs sets with the recorded *l*_PDNP_.
  (iv). We calculated length dissimilarity between Nanog LERs and H3K4me3 SERs as follows: the dissimilarity score is the area bounded by the length distribution curves of Nanog LERs and the H3K4me3 SERs.
  (v). We selected Nanog LERs from different Nanog LERs sets and calculated the above dissimilarity score. We selected the Nanog LERs set with the minimum dissimilarity score as the optimized Nanog LERs set and outputted the LERs.

We similarly called Oct4 LERs.

#### Integrating H3K4me3 SERs, H3K27me3 LERs, Oct4 LERs and Nanog LERs in mouse ES cells

We integrated all H3K4me3 SERs, all H3K27me3 LERs, the top 1000 Oct4 LERs (~ top 5% LERs) and the top 1000 Nanog LERs (~top 5% LERs) by their locations on the genome. A Nanog LER (or an Oct4 LER) overlapped by or located within 10 kb of an H3K4me3 SER was recognized as an H3K4me3-associated LER. If more than one Nanog (or Oct4) LERs were associated with an H3K4me3 SER, the LER with the highest rank was picked. An H3K27me3 LER overlapping by or located within 1 kb of an H3K4me3 SER was recognized as an H3K4me3-associated LER.

#### Cell culture

V6.5 ESCs (the F1 hybrid of 129SvJae/C57BL/6) were cultured on gelatin-coated (Millipore) plates in Dulbecco’s modified Eagle’s medium (DMEM) supplemented with 15% FBS, 0.1 mM b-mercaptoethanol, 2 mM L-glutamine, 0.1 mM nonessential amino acid, 1000 U/ml recombinant leukemia inhibitory factor (LIF; Millipore), and 30 U/ml penicillin/streptomycin. The media were changed daily, and ESCs were split every two days. HEK293T cells were cultured in DMEM with 10% FBS and 1000 U/ml penicillin/streptomycin.

#### Whole-Transcriptome shotgun sequencing (RNA-Seq) and data processing

Total RNA was isolated from 5x10^6^ mouse ES cells with the RNeasy Plus Mini Kit (QIAGEN, 74134). Poly(A)-containing mRNA molecules were purified using 1 μg total RNA as the starting material, and the library was built following NEB. Next, the Ultra RNA Library Prep Kit was used to obtain the Illumina protocols (NEB, 7530L). The library was sequenced on an Illumina HiSeq 2500 Machine. Reads were mapped to the reference genome of mm9 using TopHat with default settings [56].

#### Lentivirus production and transduction

Lentivirus was produced through the co-transfection of the lentiviral vectors (Mouse GeCKO v2 Library from Addgene; the LentiCRISPRv2 plasmid was used for single-gene targeting) with the envelope plasmid (psPAX2) and VSV-G packaging plasmids (pMD2G; 4:3:3) into HEK293T cells using Lipofectamine 2000 according to the manufacturer's instructions. Media were changed 24 hours after transfection. The virus-containing supernatant was collected and filtered through a 0.45 μm low protein-binding membrane (SLHV033RB, Millipore) 48 hours after transfection. Transduction was performed on 24-well plates with 5x10^5^ mouse ES cells in each well. The spin-infection was performed in medium containing 8 μg/mL of polybrene at 1,800 rpm for 45 min at room temperature. Transduced cells were selected 24 hr after spin-infection under 1 μg/ml puromycin for 3 days.

#### Targeted next-generation sequencing

Genomic DNA was extracted by the phenol/chloroform method from 10 million mouse ES cells. Sequencing libraries were acquired by two rounds of PCR amplification: round 1 served to amplify the targeted locus with 23 cycles [57], and round 2 served to add P5 and P7 adapters with the index for each sample with ten cycles [57]. Amplicons from the second round of PCR were sequenced on a HiSeq 2500 (Illumina). Primer sequences are shown in the S11 Table.

#### Genomic-wide CRISPR/Cas9 negative selection genetic screen

Lentivirus of GeCKO library was transduced into mouse ES cells as described above with MOI = 0.3. After puromycin selection, 10 million cells were seeded into a 10-cm dish every generation to maintain 166x coverage of the library. A total of 10 million cells from the first generation and 10th generation were used for genomic DNA extraction for targeted sequencing of sgRNA. Additionally, 50x50 μl first-round PCR reactions were performed with 825 ng genomic DNA in each reaction to achieve 100X coverage. A 2 μl volume of mixed first-round PCR products were used as templates for the second round PCR to generate targeted sequencing library.

#### Prioritizing genes that contributed to ES cells proliferation from a genomic-wide negative selection genetic screen

We prioritized genes that may contribute to mouse ES cells proliferation by the following steps:

1. We counted reads of sgRNAs in the sgRNAs pool after targeted next-generation sequencing; we only counted the reads covering 100% of the sgRNA sequence.
2. We filtered the non-targeting sgRNAs (NT-sgRNAs) in the GeCKO v2 Library and selected the sgRNAs with reads>10 at the 1st generation (a total of 874 sgRNAs) as a control pool.
3. We ranked the NT-sgRNAs in the 1st generation by their read number in descending order. We ranked them at the 10th generation in the same way.
4. We compared the reads of every sgRNA with the reads of NT-sgRNAs in the 1st generation; we calculated the corresponding ranking position of every sgRNA inside the ranked NT-sgRNA. We calculated the corresponding ranking position of every sgRNA in the 10th generation in the same way.
5. We calculated the difference in ranking position of every sgRNA between the 10th generation and 1st generation.
6. We selected the sgRNA which has the highest value of the difference from the sgRNAs set of a target gene. We used the value to rank the targeted genes in descending order.

#### Alkaline phosphatase (AP) Staining

ESCs transduced with lenti-sgRNA were first selected under 1 ug/ml puromycin for three days. AP staining was performed according to the manufacturer’s instructions for the Alkaline Phosphatase Detection Kit (SCR004, Millipore).

#### Counting indel types of a targeted locus by CRISPR/Cas9

FastQ reads of targeted NGS sequencing were mapped to the target locus using Bowtie2 with default parameters apart from an adjustment to relax the gap extension penalty (option:—rdg 5,1) [58]. The CIGAR string was extracted from SAM format files for frequency counting of indel types. Primer sequences are listed in the S11 Table.

#### Vectors and mutagenesis

The lenticrispv2 plasmid was purchased from addgene; cDNA of Zmynd8 (MC202682) and Fam60a (MC203541) were purchased from Origene and subcloned into pyCAGIP with an additional Flag tag sequence at the C-terminus; Abt1 cDNA was directly cloned from mouse ES cells and subcloned into pyCAGIP with a Flag tag at the C-terminus.

Silent mutations against sgRNA targeting were acquired by Q5 Site-Directed Mutagenesis Kit (NEB) according to the manufacturer’s instructions on pyCAGIP[59].

The sequences of the mutant sites are as follows:

Abt1-copy2:CTACTCGGCCAAATTCCAGT***TGG***

Fam60a-copy2: CTACAGTAACCAGTCGGACG***AAG***

Zmynd8-copy2: ATTCCAGAAGCCTGTCCCCT***TAG***

#### *In Vitro* CRISPR/Cas9 cleavage assay

sgRNAs were expressed from the LentiCRISPv2 plasmid according to the manufacturer’s instructions for the HiScribe T7 High Yield RNA Synthesis Kit (NEB #E2050). Cas9 Nuclease (M0386, NEB) was used to perform *in vitro* cleavage on the same amount of input plasmid. After overnight digestion, samples were resolved on a 0.8% agarose gel with 10 kb DNA Ladder (B600032, Sangon Biotech).

#### *In vivo* CRISPR/Cas9 cleavage assay

The same amount of plasmids were transiently transfected into wide-type and CRISPR/Cas9-expressing mouse ES cells. At 24 hours after transfection, the cells were harvested and subjected to Western blotting analyses.

#### Antibodies for Western blotting analyses

Cells were harvested and lysed in SDS-PAGE sample buffer. Equal amounts of total protein were loaded in each lane. Proteins were resolved by SDS-PAGE, transferred to 0.45 μm PVDF membranes, and probed with the indicated antibodies. The antibodies used for Western blotting were as follows: anti-α-tubulin (Sigma DM1a) and anti-Flag (a gift from Huang Lab).

#### Narrow down gene prioritization list of the genomic-wide negative selection genetic screen by SICER and MUSIC

To search the minimum candidate genes, including Zmynd8, Abt1, and Fam60a, from the SICER results, we filtered genes with the following criteria: 1. marked by broad H3K4me3 E-signals; 2. marked by top 11899 Nanog or top 11899 Oct4 broad E-signals; 3. not marked by broad H3K27me3 E-signals. Then, we overlapped the candidate genes with the gene prioritization list of the genomic-wide negative selection genetic screen. We selected genes with higher or equal ranks compared to Zmynd8, Abt1, and Fam60a in the gene list. These were the genes narrowed down by SICER from the genomic-wide negative selection genetic screen.

We used the same strategy to get the genes narrowed down by MUSIC from the gene prioritization list of genomic-wide negative selection genetic screen. However, the genes were marked by top 2043 Nanog or top 2043 Oct4 broad E-signals and not by top 11899 Nanog or top 11899 Oct4 broad E-signals.

## Supporting information

S1 Fig The plot of read signals of input datasets with 1×PCR and 3×PCR amplification at a genomic region. The input datasets are used as controls for ERs calling in the 1×PCR and 3×PCR Nrsf ChIP-Seq datasets of the Pfsk cell line.
(EPS)

S2 Fig CLUES and MACS2 reliable-ERs-rate curves of 12 3×PCR Nrsf datasets. (EPS)

S3 Fig CLUES and MACS2 reliable-ERs-rate curves of 19 low SNR 1×PCR datasets studied in Fig 2C. (EPS)

S4 Fig CLUES and MACS2 reliable-ERs-rate curves of 13 datasets in which MACS2 detects fewer than half ERs of CLUES at a default q<0.05. The reliable-ERs-rate curves of CLUES with a q<0.05 and MACS2 with a q<0.05 and q<0.95 have been plotted.
(EPS)

S5 Fig The plot of read signals of different datasets. a. Reads signals in Chromosome 1 for two input samples from mouse ES cells. The number of ERs detected by MACS2, CLUES, MUSIC, SISSRs and PeakRanger in whole genome is listed.
b. Reads signals in chromosome 1 for an over-amplified input sample of HEPG2 cell line and a normal input sample of HEPG2 cell line. The number of ERs detected by MACS2, CLUES, MUSIC, SISSRs and PeakRanger in whole genome is listed.
c. Reads signals in Chromosome 1 for H3K27me3 ChIP-Seq data and input from mouse ES cells. The number of ERs detected by MACS2 and CLUES in the whole genome is listed.
d. Reads signals in the genome region of Hoxa family for the H3K27me3 ChIP-Seq sample and the input sample from mouse ES cells. The ERs detected by CLUES and MACS2 are shown.
(EPS)

S6 Fig The median length of the top 1000 broad E-signals identified by CLUES, MUSIC, and MACS2 from 105 H3K4me3 datasets sorted alphabetically. (EPS)

S7 Fig Comparing the integrity of the top 1000 broad E-signals identified by CLUES, MACS2, and MUSIC from 105 H3K4me3 datasets. The multiple-rate is the percentage of a given method's top 1000 broad E-signals detected as multiple E-signals by its rival. The fragment rate is the percentage of the given method's top 1000 broad E-signals detected as fragmented E-signals by its rival.
(EPS)

S8 Fig The number of GO terms from top 1000 broad H3K4me3 E-signals identified by CLUES, MUSIC, and MACS2 from 105 H3K4me3 datasets. (EPS)

S9 Fig The reciprocal coverage of GO terms from MUSIC and CLUES broad H3K4me3 E-signals. A. In 85% of datasets, more than 20% of GO terms from the top 100 MUSIC broad H3K4me3 E-signals overlap with GO terms from the top 100 CLUES broad H3K4me3 E-signals. A total of 93 H3K4me3 datasets were used.
B. In 94% of datasets, more than 80% of GO terms from the top 100 MUSIC broad H3K4me3 E-signals overlap with GO terms from the top 1000 CLUES broad H3K4me3 E-signals. A total of 93 H3K4me3 datasets were used.
C. In 10% of datasets, more than 50% of GO terms from the top 100 CLUES broad H3K4me3 E-signals overlap with GO terms from the top 1000 MUSIC broad H3K4me3 E-signals. A total of 105 H3K4me3 datasets were used.
(EPS)

S10 Fig The characteristics of the top 1000 broad E-signals identified by CLUES(C), MUSIC(M), PeakRanger(P) and SICER(S) from 26 H3K27me3 and 34 H3K36me3 datasets. The total length (Genome coverage), minimum reads-enrichment (Enrichment), the number of covered genes (Gene-rate) and the number of broad E-signals without genes (Off-target rate) are compared. Higher genome coverage, higher enrichment, higher gene-rate or lower off-target rate reflects the better performance of a method. The heat-maps are rank-ordered based on the first letter of their name from A to Z.
(EPS)

S11 Fig The top GO terms from 690 top-ranked genes revealed by a CRISPR/Cas9 negative selection genetic screen. (EPS)

S12 Fig The genes revealed by the integrated analysis of MUSIC and SICER are not enriched at the top of the list from a CRISPR/Cas9 negative selection genetic screen (Kolmogorov–Smirnov test). (EPS)

S13 Fig The plots of broad E-signals of H3K4me3, H3K27me3, Nanog and Oct4 and RNA-Seq signals at Fam60a, Abt1, and Zmynd8 locus. Y-axes, RPKM of Nanog, Oct4, H3K4me3, and H3K27me3 ChIP-Seq datasets and RNA-Seq datasets.
(EPS)

S14 Fig The slower proliferation of mutant ES cells with Fam60a, Zmynd8 or Abt1 knockout can be partially restored by re-expression of the corresponding gene with a silent mutation that prevents sgRNA targeting (labeled with a star). The graph plots the percentages of mutant ES cells normalized against wild-type ES cells. Error bars indicate the SD of triplicates.
(EPS)

S15 Fig Indel percentage of Fam60a, Zmynd8, and Abt1 loci after CRISPR-sgRNA targeting. Each panel shows Indels from one locus. The first line is the reference sequence with a framed PAM sequence and an underlined sgRNA sequence.
(EPS)

S16 Fig Silent mutations of Fam60a, Zmynd8, and Abt1 resistant to corresponding sgRNA targeting *in vitro*. Controls, plasmids carrying wild-type genes.
(EPS)

S17 Fig The estimation of shift parameter in CLUES. a. A diagram is showing converting sorted and neighboring reads into bins on a chromosome region.
b. Length distribution of the bins of a Ctcf ChIP-Seq dataset under different shift parameters is plotted. The shift parameter under which the curve has the most short-bins, which are bins on the left side of the peak in the plot (bin length < 2 bp), are selected as the shift parameter (60 bp here). The X-axis is the bin length, and the Y-axis is the number of bins.
c. Shift parameters of 22 ENCODE Ctcf datasets estimated by MACS2 and CLUES. The datasets are sorted according to the first letter of their names.
(EPS)

S1 Table The parameters and thresholds of CLUES. (XLSX)

S2 Table The human and mouse ChIP-seq data used in this work. (XLSX)

S3 Table The commands used by the methods to identify ERs and broad E-signals from ChIP-Seq datasets. (XLSX)

S4 Table The ratio of reliable-ERs-rate curves between CLUES and the other methods. (XLSX)

S5 Table The number of genes associated with top 100 broad H3K4me3 E-signals prioritized by CLUES, MACS2, and MUSIC. (XLSX)

S6 Table GO terms of the top 100 broad E-signals identified by CLUES, MUSIC and MACS2 from H3K4me3 ChIP-seq datasets of H7esDiffa14d. (XLSX)

S7 Table The number of GO terms and genes associated with the top 100 broad E-signals identified by CLUES and other methods in mouse ES cell ChIP-Seq data of H3K4me3, H3K27me3, Nanog and Oct4. (XLSX)

S8 Table GO term of the top 100 broad E-signals identified by CLUES and other methods from mouse ES cell ChIP-Seq datasets of H3K4me3, H3K27me3, Nanog and Oct4. (XLSX)

S9 Table Genes with top-ranked broad E-signals of H3K4me3, Nanog/Oct4 and without H3K27me3 broad E-signals. (XLSX)

S10 Table Motif profiles of Ctcf and Nrsf. (XLSX)

S11 Table The primer sequences of targeted next-generation sequencing. (XLSX)
