# Correction: Clustering-local-unique-enriched-signals (CLUES) promotes identification of novel regulators of ES cell self-renewal and pluripotency

**DOI:** 10.1371/journal.pone.0210042

**Published:** 2018-12-27

**Authors:** Chao Wu, Yang Jiao, Manli Shen, Chen Pan, Guo Cheng, Danmei Jia, Jing Zhu, Long Zhang, Min Zheng, Junling Jia

In the Funding section, the grant number from the funder the Zhejiang Provincial Natural Science Funds is listed incorrectly. The correct grant number is: LR15C060001.
